# Light Intensity and Wavelength Modulate Antioxidant Secondary Metabolites in Embryonic Axis of Chickpea Sprouts

**DOI:** 10.3390/foods15142578

**Published:** 2026-07-22

**Authors:** Luis F. Pérez-Hernández, Robert Winkler, Marco A. Mata-Gómez

**Affiliations:** 1 Tecnologico de Monterrey, School of Engineering and Sciences, Ave. Eugenio Garza Sada 2501 Sur, Col: Tecnologico, Monterrey 64700, Nuevo Leon, Mexico; a01732083@tec.mx; 2 Unidad de Genómica Avanzada, Cinvestav, Km. 9.6 Libramiento Norte Carr. Irapuato-León, Irapuato 36824, Guanajuato, Mexico; robert.winkler@cinvestav.mx

**Keywords:** *Cicer arietinum* L., germination, metabolic pathways, nutrition, seeds

## Abstract

Chickpea (*Cicer arietinum* L.) is a highly nutritious legume with significant potential as a food plant. With the advent of climate change and the challenge of feeding a growing population, strategies to produce high-nutrient crops are of utmost importance. In this context, this study describes how light intensity and wavelength affect the embryonic axis of chickpea sprout metabolism, using untargeted metabolomics. Chickpea sprouts were grown under varying light wavelengths (red—650, green—550, and blue—450 nm) at two intensities (75 and 275 µmol·m^−2^·s^−1^). Analyses were restricted to the embryonic axis (hypocotyl), excluding the cotyledons, which are a reserve-rich tissue. Results showed that high-intensity red light (RH) increased phenolic content by more than 300% compared to controls. Green and blue light treatments significantly increased the protein content by more than twice that of the dark control. Antioxidant activity was significantly higher in sprouts grown under high-intensity blue light (BH). Additionally, the results suggested that pathway regulation is affected not only by wavelength but also by light intensity, with greater significance and effect under BH and RH treatments in isoflavonoid biosynthesis. High quercetin concentration found under BH is hypothesized to explain the high antioxidant activity when compared to the rest. For instance, these findings highlight the potential of light manipulation to modulate and enhance the nutritional and functional qualities of chickpea sprouts’ embryonic axis, contributing to food security and human health. Further studies need to be conducted to answer whether these metabolite changes directly translate to the nutritional quality of the whole edible sprouts.

## 1. Introduction

The global food system faces two major challenges: feeding a growing population while promoting both human health and mitigating climate change. The current human population is around 8.2 billion, and it is predicted to reach 9.7 billion by 2050 and 10.4 billion by the end of the century [1]. This increase in the human population will lead to high demand for high-quality, health-promoting food. Consequently, it is vital to identify crop management strategies that preserve or enhance the nutritional quality and bioactive compounds of plants under climate-stressed conditions, while shrinking agriculture’s footprint.

Sunlight is crucial so that plants can carry out photosynthesis, produce nutritious biomass, and produce compounds of interest, ultimately working as a food source for humans. Nonetheless, climate change can affect light availability, intensity, and quality. Notably, atmospheric composition can be altered by climate change, affecting the scattering and absorption of specific wavelengths of light or causing overexposure to light in areas with ozone depletion. This will, in turn, affect metabolite pathways, which are highly important for maintaining the nutritional content and functional properties of crops. In this scenario, studying how light intensity and quality affect crop nutrition is crucial to understanding which pathways are affected and to what extent. This would provide valuable information to further develop strategies (i.e., breeding of engineering plants that are more resilient to changes in light) to ensure or even enhance the nutritional content of crops. Moreover, light manipulation in controlled environments, such as vertical farming or greenhouses, can be a straightforward means of inducing plant growth towards sustainable agricultural practices.

Hereby, chickpea (*Cicer arietinum* L.), also known as “garbanzo bean,” was selected as a crop model to understand the relationship between light and metabolite pathways. This pulse is one of the most relevant legumes in human society, just after field pea (*Pisum sativum* L.) and common bean (*Phaseolus vulgaris* L.) [2,3,4]. Its cultivation and consumption are widespread globally due to its high protein content and quality. Chickpeas are highly relevant in Mediterranean and Middle Eastern cuisines, as well as in Mexico, where they are used in dishes like hummus and falafel, and are also widely used in Indian cuisine for salads, soups, stews, and curries [2,5].

Seed germination has been identified as a strategy for enhancing nutritional composition in legumes. The sprouting process has been reported to improve protein digestibility, increase certain secondary metabolites of interest, and reduce antinutritional factors, therefore yielding products with higher functional value than raw seed [5].

It is known that the nutritional composition of chickpeas is affected by various physical conditions [5], such as water availability [6], heat [3,7], and light [4,6]. Nonetheless, how light intensity and wavelength combination affect metabolic pathway activation, and consequently the secondary metabolite expression of chickpeas, remains unclear. Previous research has suggested that relevant compounds for human health, i.e., biochanin A, are increased under RGB LED light compared to sunlight and total darkness, with higher concentrations in 7-day-old chickpea sprouts [8]. Beyond this, it has been reported that blue light plays a significant role in the synthesis of kaempferol in lentils, a compound with high antioxidant activity [9].

Data on how light quality affects chickpea secondary metabolism remain fragmentary, since most experiments use a single red–blue combination, neglect the green/yellow region, and focus mainly on morphological traits [10]. Most of the studies vary in color but rarely in intensity, analyzing specific compounds by targeted methods. Some studies have examined green light; nevertheless, intensity has not been actively explored, and the whole sprout is usually analyzed [2,8]. In contrast, this study aims to investigate whether light influences the secondary metabolite accumulation of chickpea sprouts as a model grown under controlled environments with varying light wavelengths and intensities. The effects of light on chickpea metabolomics, morphology, physiology, and antioxidant characteristics at three wavelengths and two intensities were studied. Germinated seeds (sprouts) are themselves recognized as a strategy for improving the nutritional value of legumes; therefore, these findings will help to develop strategies to further enhance antioxidant secondary metabolite accumulation in chickpea towards sustainable agriculture.

## 2. Materials and Methods

### 2.1. Chemicals and Reagents

Bradford reagent (23200), Bovine Serum Albumin (BSA) Standard Ampules, 2 mg/mL (23209), Anti-Anti antibiotic–antimycotic solution (11570486), methanol LC-MS grade (615130025), water LC-MS grade (047146.K2), and formic acid LC-MS grade (28905) were obtained from Thermo Fisher Scientific (Waltham, MA, USA). Gallic acid (G7384), Folin–Ciocalteu phenol reagent (F9252), Trolox antioxidant standard (93510), ABTS (A1888), potassium persulfate (216624), sodium carbonate (451614), acetone (270725), hydrochloric acid (7647010), and absolute ethanol (64175) were bought from Sigma-Aldrich (St. Louis, MO, USA). Ultrapure water was produced with a Milli-Q Integral 15 system (Merck Millipore, Burlington, MA, USA). Chickpea seeds (*Cicer arietinum* L., cultivar UC-27; seed lot sd003010) were obtained from L.A. Hearne Company (King City, CA, USA). The germination substrate consisted of peat moss (144085) and perlite (152537) sourced from Pisumma Company (Mexico City, Mexico). All chemicals were used without further purification. Greenhouse monitoring was conducted using the following sensors: temperature and humidity (DHT11, Aosong Electronics Co., Ltd., Guangzhou, China), Capacitive Anticorrosive Soil Moisture (AR1188, UNIT Electronics, Mexico City, Mexico), and light (TSL2561, ams-OSRAM AG, Premstätten, Austria).

### 2.2. Plant Material and Sprouting Growth

**Figure 1:**
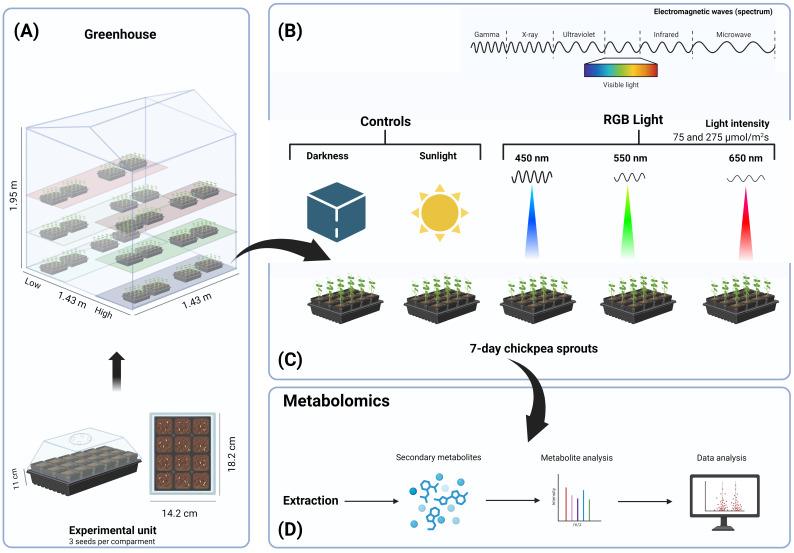
Experimental setup for chickpea growth in a greenhouse during a seven-day germination period. (**A**) Experimental unit containing twelve compartments and a schematic representation of the greenhouse with its dimensions. Experimental units containing seeds, depicted on the left and right shelves, were exposed to low (75 µmol·m^−2^·s^−1^) and high (275 µmol·m^−2^·s^−1^) light intensities. Experimental units are located above shelves, which avoids the light mix. (**B**) Full experimental design where one run is represented (three replicates were performed), two intensities are used as well as two controls, one positive (Control Sun, CS) and the other negative (Control Dark, CD). (**C**) Chickpea sprouts grown after 7 days, ready for sample preparation. (**D**) Workflow for metabolomics from sample extraction to data analysis.

The experiment was carried out in Puebla City, Mexico (19°01′05.0″ N 98°14′32.4″ W), during the months from March to May (2024–2025). Sprout material from both growing seasons was pooled by treatment. Environmental conditions reported, including sunlight intensity, were contrasted with those from the Mexican government in Puebla City, with an average temperature of 20 °C during the experimental period (Figures S1 and S2). Briefly, chickpea seeds were soaked in distilled water to remove superficial contaminants. The next step involved selecting viable seeds based on their buoyancy [11]. After selecting the chickpea seeds, they were carefully washed and soaked overnight in a 100× solution containing Anti-Anti (1 µL), prepared in 1 mL of Milli-Q water. Viable seeds were assigned to the pots and compartments randomly. Concomitantly, the order in which treatments were sown was varied across the replicates to avoid any systematic effect of handling sequence. The sprouting growth was carried out in a self-designed-and-built greenhouse, depicted in Figure 1. Greenhouse dimensions and characteristics are presented in Figure 1A as well as in Figure S3. Temperature (±2 °C) and relative humidity (±5%) conditions were monitored throughout the day using a DHT11 sensor, which was connected to an Arduino microcontroller (see Code S1). An RGB LED system (Tecnoled, ML-REF-RGB), with nominal peak wavelengths (λ_peak_) of Red (≈650 nm), Green (≈550 nm), and Blue (≈450 nm), was set inside the greenhouse. In parallel to greenhouse building and setting, plastic pots containing peat moss (80% *w*/*w*) and perlite (20% *w*/*w*) were prepared; the substrate had a pH of 6.7 ± 0.1 (mean ± SD, *n* = 4 independent subsamples and *n* = 3 technical replicates), determined in a 1:5 (*v*/*v*) suspension in deionized water. The equipment used for pH determination was a benchtop pH meter (PH550, Oakton Instruments, Vernon Hills, IL, USA; accuracy ±0.01 pH). Soil humidity was set to 60% of field capacity; semi-closed pots (0.182 mL × 0.142 mW × 0.11 mH) with 12 compartments were used to maintain optimal relative humidity (50%). A total of 36 seeds per pot were then planted at a depth of 1 cm, meaning 3 seeds per compartment. For the experiment, a total of 864 seeds were used (6 light treatments and 2 controls). The total number of seeds was calculated as 8 [treatments and controls] × 3 replicates × 36 [12 compartments multiplied by 3 seeds per compartment].

Pots containing the seeds were then placed inside the greenhouse, and experiments were carried out as schematized in Figure 1B,C. The intensities were set to 75 µmol·m^−2^·s^−1^ (Low) and 275 µmol·m^−2^·s^−1^ (High). Chickpea sprouts were also grown under natural sunlight and total darkness as positive and negative controls, respectively. Moreover, the photoperiod was 16 h of light and 8 h of darkness [12]. Light regulation was automated using a timer (TEMP-08E, Steren, Mexico City, Mexico) and was constantly monitored to ensure the photoperiod was accurate. Then it was set to 8 light treatments: Red High (RH), Red Low (RL), Green High (GH), Green Low (GL), Blue High (BH), Blue Low (BL), and two controls: Control Sun (CS) and Control Dark (CD). The average temperature was 26 °C, with maximum and minimum temperatures of 32 °C and 15 °C, respectively. Experimental units were isolated by light-blocking material, preventing any light mixing that could disturb the treatments (Figures S3 and S4). The sprouting procedure was conducted in triplicate; fresh weight and sprout length were recorded, while the germination rate was determined as follows:

(1) $$Germination\;rate\;\left(\%\right)=\left(\frac{Number\;of\;germinated\;seeds}{Total\;number\;of\;seeds}\right)\times 100$$

After 7 days, samples were collected and stored at −80 °C until further use (including the metabolomics workflow shown in Figure 1D).

### 2.3. Morphological Analysis

After seven days of growth, sprouts were harvested, photographed, and analyzed for their morphology. RizhoVision Explorer (2.0.3) was used to evaluate the effects of light on the structural composition and morphological traits of chickpea sprouts.

Images (3024 × 4032 pixels) were fed into the software and processed according to [13,14]. Then, to evaluate the light effect, the following parameters were determined: Total Root Length (cm), Volume (cm^3^), Depth (cm), and Number of Root Tips. Accordingly, it was necessary to estimate the number of Pixels Per Millimeter (PPM) for software-based calculations. This was performed using the following equation:

(2) $${PPM}_{new}=\frac{K}{{TRL}_{measured}}$$

where *K* is a constant that represents the *Total Root Length* (*TRL*) in pixels and is calculated as:

(3) $$K={TRL}_{software}\cdot {PPM}_{initial}$$

with ${TRL}_{software}$ representing the root length measured by the software, in mm, using the initial PPM value in the program. The value of ${TRL}_{measured}$ corresponds to the real value of the root length obtained from previous measures in mm. Finally, to assess morphological changes due to light exposure, the Lateral-Root Branching Density (LRBD) was calculated as described by ref. [15]. The equation is as follows:

(4) $$LRBD=\frac{Number\;of\;root\;tips}{{TRL}_{measured}}$$

### 2.4. Sample Preparation

Chickpea sprouts were harvested after seven days of growth. From these sprouted chickpea seeds, only the stem was used for further analysis. The stem was defined as the embryonic axis composed mainly of the hypocotyl and the radicle. This specific tissue was selected because it exhibits faster metabolomic reprogramming than the cotyledons, and sprouts have been shown to have high nutritional value [16]. Additionally, this tissue was selected because the cotyledons are primarily reserve-dominated. The composition of the cotyledons is primarily starch and storage proteins, specifically globulins [17]. This means that including the cotyledons in the homogenized sample will significantly affect the analysis due to the biomass proportion of the embryonic axis and the cotyledons, which will ultimately hide the light effects on the metabolic germination process. The material obtained was stored at −80 °C and subsequently lyophilized (FreeZone 4.5, Labconco, Kansas City, MO, USA). The dried material was pulverized and stored at −20 °C for subsequent steps.

### 2.5. Phenolic Extraction and Quantification

Phenolic extraction process from lyophilized chickpea sprouts was performed using a solvent extraction composed of acetone/water/hydrochloric acid (70:29:1, *v*/*v*/*v*). For extraction, the vegetal material was mixed with the extraction solvent in a 1:50 ratio. The mixture was agitated at 300 rpm for three hours in an orbital shaker (Orbit 4, Labnet International, Edison, NJ, USA) at room temperature. Additionally, the mixture was left overnight for an extra 17 h. The sample was then centrifuged at 6018× *g* for 10 min (Eppendorf 5702 R centrifuge, Eppendorf, Hamburg, Germany). The supernatant was separated into a new tube. The process was repeated to extract total phenolic compounds, and the supernatant from the second extraction was combined with that from the first. The final extract was stored in the dark at 4 °C within 2 days as previously described by [18].

The Folin–Ciocalteu method was used to determine the TPC as described earlier [18,19]. Gallic acid (10–100 µg/mL) was used as a standard to create the calibration curve. A sample aliquot of 20 µL was placed into a 96-well microplate along with 10 µL of Folin’s reagent. The mixture was shaken slowly. After 3 min, 30 µL of 10% *m*/*v* sodium carbonate (NaCa_3_) was added, and the mixture was incubated for 2 h at room temperature. Finally, after incubation, the absorbance of the mixture was measured at 765 nm using a microplate reader (BioTek Citation 5, Agilent BioTek, Winooski, VT, USA).

### 2.6. Antioxidant Activity Assay

The 2,2-Azinobis (3-ethyl-benzothiazoline-6-sulfonic acid (ABTS) assay was used to determine the antioxidant activity of the samples. An ABTS^+^ stock solution was initially prepared by mixing ABTS (7 mmol/L) with potassium persulfate (K_2_S_2_O_8_) (2.45 mmol/L). The solution was then stored in total darkness at room temperature for 12 to 16 h prior to usage. After that, the ABTS stock solution was diluted with 50% *v/v* ethanol to adjust the absorbance at 734 nm to 0.75–0.80.

For the assay, in a 96-well plate, 10 µL of the phenolic sample was combined with 190 µL of the diluted ABTS. After adding the ABTS, samples were incubated for 10 min at room temperature in total darkness. The absorbance resulting from the mixture incubation was measured at 734 nm in a microplate reader [20].

The ABTS^+^ scavenging capacity of the chickpea samples was quantified using the Trolox equivalent antioxidant capacity (TEAC). Concentrations of Trolox in the standard curve varied from 0 to 200 mg/L. To construct the Trolox standard curve, the percentage of inhibition was plotted against Trolox concentration at 734 nm. The inhibition percentage of ABTS^+^ was calculated as follows:

(5) $${Inhibition}_{sample}\;\left(\%\right)=\;\frac{{Abs}_{control}\;-\;{Abs}_{sample}}{{Abs}_{control}}\cdot 100$$

where *Abs_control_* is the absorbance of the control and *Abs_sample_* is the absorbance of the sample. To obtain the TEAC, the percentage of inhibition was used in conjunction with the Trolox standard curve.

### 2.7. Protein Extraction and Quantification

Protein extraction was performed under alkaline conditions. Briefly, a solution of 1M NaOH (100 µL) was added to the lyophilized chickpea sprout (10 mg). Extraction was performed for 20 min. Subsequently, the supernatant was collected and stored for further analysis. Protein quantification was conducted using the Bradford method [21]. A volume of 250 µL of Bradford reagent was mixed with 5 µL of the sample in a 96-well microplate. The plate was agitated for 30 s. Afterward, the incubation was performed in total darkness for 10 min, and absorbance was measured at 595 nm to determine protein content [22]. A BSA solution (2 mg/mL) was used as a standard to construct the calibration curve (125–2000 µg/mL) and to determine the protein content in the sample.

### 2.8. LC-MS

Metabolite extraction was performed by mixing 0.1% (*w*/*v*) sample in a methanol–water solution (80:20, *v*/*v*). The mixture was vortexed for 1 min and subsequently sonicated for 30 min in a cold ultrasonic water bath (Branson 5510, Emerson Electric Co., Brookfield, CT, USA). Then, samples were stored at 4 °C until precipitation was observed. The samples were centrifuged at 15,890× *g* for 5 min at 4 °C, and the supernatant was dried under a nitrogen stream. The dried extracts were reconstituted by adding 200 µL of methanol before LC-MS analysis. LC-MS was performed in an Agilent HPLC system (Agilent Technologies, Santa Clara, CA, USA) coupled with an ODS-2 Hypersil column (250 × 4.6 mm, 5 µm, 31605-254630, Thermo Fisher Scientific, Waltham, MA, USA) for chromatographic separation. A volume of 10 µL was injected into the system, and separation was performed at a flow rate of 300 µL/min with the column temperature set to 30 °C. The mobile phase consisted of solvent A (0.1% formic acid in water) and B (methanol). The gradient used was as follows: 0 min: 20% B, 10 min: 100% B, 12 min: 100% B and 15 min: 20% B. MS was performed with the following conditions: positive mode, full-scan ITMS (ion-trap mass spectrometry) mass range 50 to 2000 *m/z*, capillary voltage 15 V, capillary temperature at 280 °C, tube lens voltage at 65 V, sheath gas flow rate at 30, and spray voltage at 5 V. The MS analyzer used was a 3D Quadrupole Ion Trap (LCQ Fleet), Thermo Finnigan (LCF10500, Thermo Fisher Scientific, San Jose, CA, USA). For each condition, the three biological replicates were integrated into one sample before injection (*n* = 1 per condition), and a methanol (MeOH) blank was included for background subtraction.

### 2.9. MS Data Processing

Computational workflow and metabolomics data processing were performed on a CPU (8 cores, 16 threads) with a Zen 3 microarchitecture (Ryzen 7 5700G), 64 GB of DDR4-3200 MHz RAM, running Windows 11 (64-bit). For metabolomics analysis, the raw mass spectrometry data files were first converted using ProteoWizard version 3.0.25073 into a format suitable for further analysis (from .raw to .mzML) as described elsewhere [23]. The converted files were then imported into RStudio (v4.4.2) for handling spectrometry data. The processing was implemented using the workflow proposed elsewhere [24,25]. Peak detection used XCMS CentWave (ppm = 350; peak width 7–37 s; signal-to-noise threshold = 5; prefilter = 3 scans/100 intensity; mzdiff = 0.0089; noise = 0); the process was performed from 60 s to 900 s. Retention time correction was performed using Obiwarp (binSize = 0.5), and peak grouping was implemented with the peak-density method (bw = 0.7; binSize = 0.25; minFraction = 0.2). Missing values were first addressed by gap filling using fillChromPeaks and then by imputation of the remaining missing values at an estimated limit of detection derived from the lower-quantile tail of the log_2_-intensity distribution. Blank subtraction was performed using the .mzML file from methanol (MEOH) for every sample. Normalization was performed by sample median scaling (each sample scaled to a common reference median) and log_2_-transformed. Main libraries in R were XCMS and Msnbase [25]. The parameters were initially adjusted using the Isotopologue Parameters Optimization (IPO) approach, as described by ref. [26]. The final parameters were adjusted manually. Adduct handling was performed within the Mummichog algorithm, which is based on mass-to-charge ratio (*m/z*) matching, and metabolite annotation was performed using the KEGG database. Therefore, positive mode adduct rules were applied with primary ions enforced (using as candidates [M + H]^+^, etc.). Pathway enrichment (functional analysis LC-MS) was performed using the library MetaboAnalystR and gmx_kegg (cross-referenced with the KEGG database). Analysis was performed in positive-ion mode, with a mass tolerance of 500 ppm, and the integrated Mummichog and GSEA method. Mummichog statistical parameters were *p*-value threshold (0.05, pathways were considered significantly enriched at FDR-adjusted *p* (gamma) < 0.05), minimum number of metabolites per pathway (5), enforce primary (enabled), and number of permutations (10^6^) [27,28]. It is important to point out that the identification is putative, rather than confirmed, yet it provides insight into the changes in the metabolite pathways. The scripts and .mzML files can be reviewed in the Supplementary Materials section.

### 2.10. Statistical Analysis

The information processing was carried out in RStudio. The analysis consisted of an Analysis of Variance (ANOVA), which was applied to evaluate the effect of the factors (light wavelength and intensity) on phenolic compounds, antioxidant activity, and protein contents for each treatment. A 95% confidence interval was used for processing the data. Additionally, Tukey’s Honest Significant Difference (HSD) test was performed at the 95% confidence level to compare means between treatments and controls, thereby determining significant differences among the groups. Biological samples were performed in triplicate in the case of biochemical experiments; three technical replicates were performed. For untargeted metabolomics (*n* = 1 per condition), feature-level differential abundance relative to the positive control (CS) was evaluated from log_2_ fold changes through an intensity-dependent variance model. Furthermore, the Benjamin–Hochberg procedure was used, and *p*-values were adjusted using multiple comparisons across all features.

## 3. Results

### 3.1. Red Light Increases Stem Elongation

**Figure 2:**
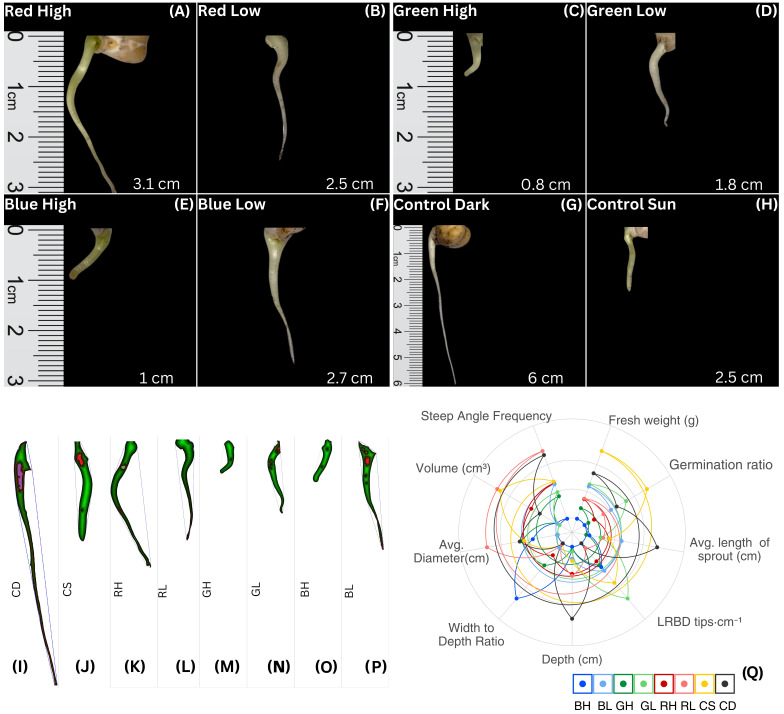
Morphological characteristics and metrics of chickpea sprouts under different light treatments. Chickpea sprouts (**A**–**H**) grown under red–green–blue light at two intensities, high = 275 µmol·m^−2^·s^−1^ and low = 75 µmol·m^−2^·s^−1^. Analysis obtained from RizhoVision Explorer (2.0.3) (**I**–**P**). Radar chart (**Q**) showing all morphological traits on a normalized scale, with the outermost circle corresponding to the maximum observed value for that trait across all treatments. The concentric grey circles provide reference steps (outer circles represent higher values), so each point represents the trait value. For example, in fresh weight (g), CS has the highest value, while BH has the lowest.

Biomass amount, germination ratio, and average length of sprout were measured to evaluate the effect of light quality and quantity on the morphological characteristics of chickpea sprouts (Figure 2 and Table S1). Biomass produced during germination did not differ significantly (α = 0.05) between treatments, except for Blue High (BH) compared with Control Sun (CS), in which BH was the lowest and CS the highest, as observed in Table S1. This can also be inferred from the sprout length results presented in Figure 2, particularly in panels H and E. Indeed, sprout length varied significantly between treatments (Figure 2A–H). Seeds grown under total darkness (CD) yielded the longest sprouts among all, with an average length of 5.77 cm ± 0.37 (Table S1 and Figure 2G). Chickpeas under 75 µmol·m^−2^·s^−1^ (Figure 2B,D,F) tend to have a longer sprout length when compared to those growing under 275 µmol·m^−2^·s^−1^ (Figure 2A,C,E). On its own, Red High treatment yielded sprouts with longer stem length than Red Low (Figure 2A,B).

The analysis performed using RizhoVision Explorer (2.0.3) (Figure 2I–P) was used to build the radar chart (Figure 2Q). In this figure, it can be observed that the highest fresh weight corresponds to the CS treatment, while the lowest corresponds to the GH treatment. Moreover, the longest axis stem corresponds to CD. Germination ratios across all treatments showed no statistically significant differences (α = 0.05).

### 3.2. RGB Light Significantly Increases the Phenolic Content, Antioxidant Activity, and Protein Content

Seeds exposed to high-intensity red light (RH) and low-intensity green light (GL) yielded sprouts containing higher phenol concentrations. On its part, blue light treatment did not differ at either intensity tested (75 and 275 µmol·m^−2^·s^−1^), and no significant difference was observed when sprouts were grown either in total darkness or under direct sunlight. Notably, the phenolic content was higher by over 300% in sprouts exposed to RH and GL compared to those sprouts grown under direct controls, which were sunlight or total absence of light (Table 1).

The ABTS assay was used to evaluate the effect of light on the antioxidant activity of the total phenolic compounds. This parameter depends not only on the quantity of phenols but also on the compounds themselves. The antioxidant activity determined in the treatments RL, GH, BL, and CD did not present a significant difference (*p* > 0.05). Likewise, the RH treatment and CS did not differ significantly in antioxidant activity. Nevertheless, it was found that the BH treatment can inhibit up to 80% of the ABTS^+•^ radical, in contrast to the inhibition of 22% and 45% corresponding to samples from sprouts grown in darkness and sunlight, respectively. In terms of Trolox equivalent antioxidant capacity (TEAC), the blue light extract at 275 µmol·m^−2^·s^−1^ was 88.9 ± 2.6, while that of the dark control was 25.9 ± 1.3 µmol TE g^−1^ DW, indicating a significant difference (Table 1). Protein quantification was performed to evaluate the effect of light on the protein content of chickpea sprouts. A significant increase in protein was observed in sprouts grown under GH treatment compared to CD, from 12.7 to 26.4 mg g^−1^ DW (Table 1). The protein content obtained from the high-intensity green light treatment was not significantly different (*p* > 0.05) from that of the low-intensity blue light treatment.

### 3.3. Light Exposure in Chickpea Sprouts Shifts Their Metabolomic Pathways

**Figure 3:**
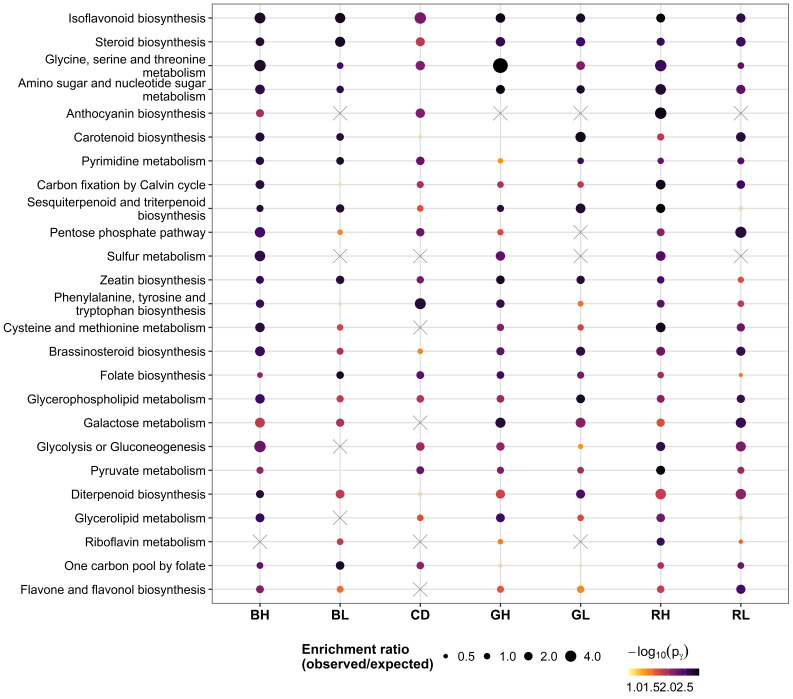
Bubble plot showing the Mummichog pathway enrichment for the top 25 metabolic pathways, selected based on the maximum combined Mummichog score (average of −log10[pγ] and −log10[empirical p]) across all treatments, where pγ denotes the pathway’s Fisher’s exact enrichment *p*-value, adjusted against a gamma distribution fitted to this null. Further, empirical p corresponds to the fraction of permutations that yield enrichment at least as significant as that observed in the data. Blue High (BH), Blue Low (BL), Control Dark (CD), Green High (GH), Green Low (GL), Red High (RH), and Red Low (RL) are the treatments corresponding to light treatments at low (75 µmol·m^−2^·s^−1^) and high (275 µmol·m^−2^·s^−1^) intensity.

Mummichog analysis was performed to evaluate which pathways were significantly enriched when sprouts were grown under the different light treatments (Figure 3). The following metabolic pathways were enriched for the treatments with the highest significance when compared to control sun (CS): glycine, serine and threonine metabolism (7.72, GH and 4.16, RH), glycolysis or gluconeogenesis (4.20, BH), anthocyanin biosynthesis (4.16, RH), phenylalanine, tyrosine and tryptophan biosynthesis (3.81, CD), isoflavonoid biosynthesis (3.69, BH). Note that the pathway name is presented along with the enrichment ratio (ER; the higher the ER, the higher the effect of the treatments on the metabolism) and the corresponding treatment using the following notation: “pathway name (ER, treatment)”. The lower enrichment ratio pathways were pyruvate metabolism (0.34, BL) and carbon fixation by the Calvin cycle (0.51, BL).

**Figure 4:**
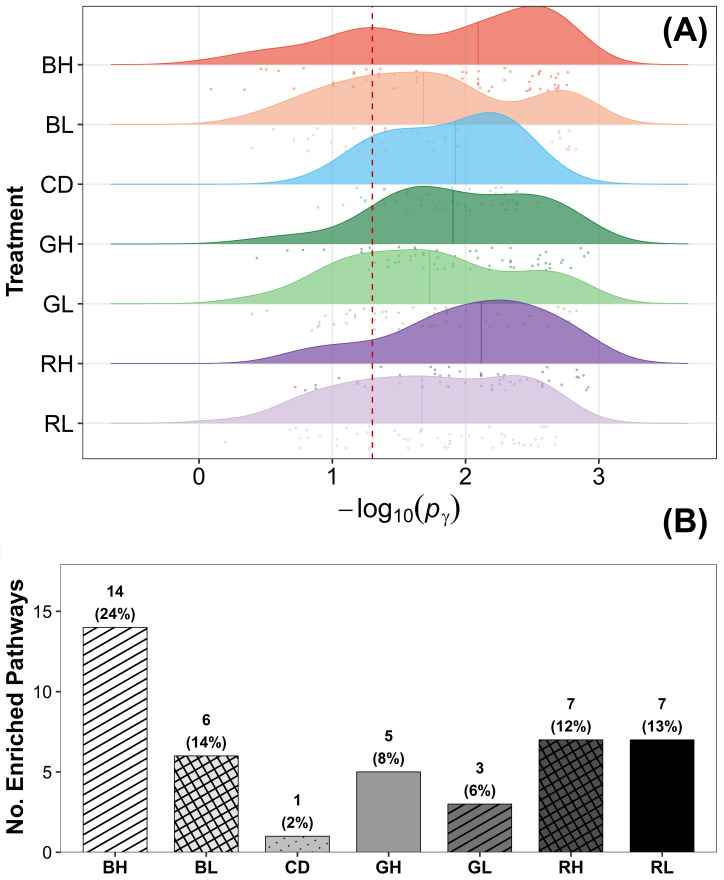
Chickpea metabolic pathways under different light conditions. Comparison of all the treatments against the positive control, Control Sun (CS). (**A**) Rain cloud plot of all the treatments. It shows one ridge per treatment; each ridge represents the kernel density estimate (KDE) of pathway significance. The jitter shows the underlying distribution, while each vertical line represents the median. The dashed red vertical line indicates the −log_10_[p_γ_] = 0.05 significance threshold. (**B**) Bar chart showing the count of the significantly enriched pathways per treatment; it only displays the pathways that meet the criteria of p_γ_ < 0.05 and empirical *p* < 0.05 at the same time. Blue High (BH), Blue Low (BL), Control Dark (CD), Green High (GH), Green Low (GL), Red High (RH), and Red Low (RL) are the treatments corresponding to light treatments at low (75 µmol·m^−2^·s^−1^) and high (275 µmol·m^−2^·s^−1^) intensity.

**Figure 5:**
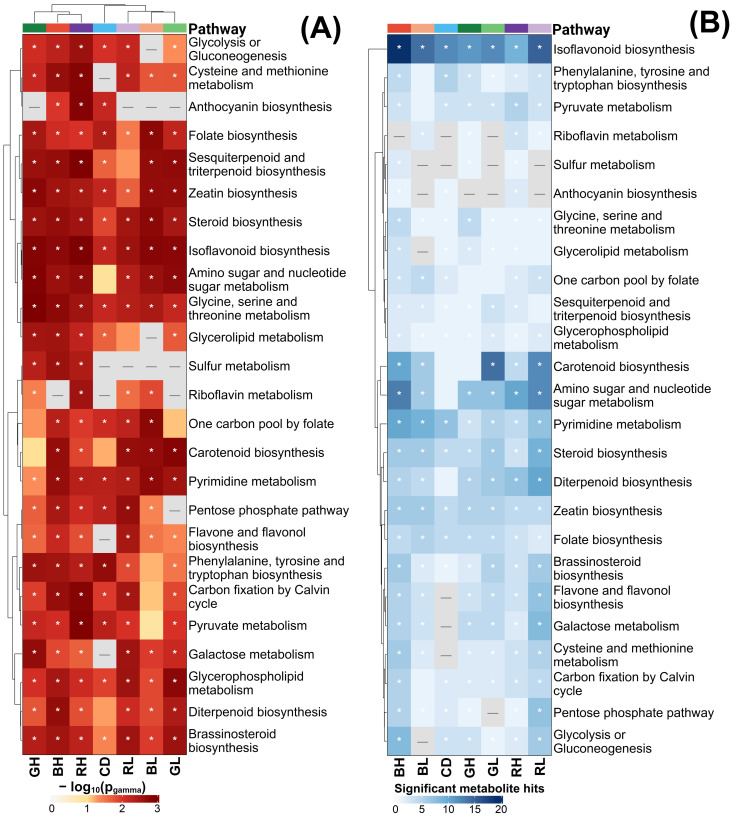
(**A**) Pathway significance heatmap of the top 25 pathways across all the treatments. The pathways (rows) and treatments (columns) were independently clustered using the Pearson correlation distance along with Ward’s D2 linkage (rows) and Euclidean distance with Ward’s D2 linkage (columns). This reveals co-enrichment; the * in each cell indicates the pathway–treatment combination with pγ < 0.05. (**B**) Heatmap with the significant metabolite hits. It displays the significant hits tentatively assigned in each treatment. The asterisk (*) indicates that the result reached statistical significance p_γ_ < 0.05. Blue High (BH), Blue Low (BL), Control Dark (CD), Green High (GH), Green Low (GL), Red High (RH), and Red Low (RL) are the treatments corresponding to low (75 µmol·m^−2^·s^−1^) and high (275 µmol·m^−2^·s^−1^) light intensity.

When comparing of all the treatments against the positive control, Control Sun (CS), the distribution differed among the treatments relative to CS, which was shifted to the right, indicating high significance, as depicted in the rain cloud plot in Figure 4A. The treatment BH showed the highest number of enriched pathways (14), while the lowest is CD (1), as shown in Figure 4B. This can also be observed in the metabolite heatmaps presented in Figure 5. From this figure, pathway enrichment analysis not only showed extensive metabolic modulation across all the treatments, but also showed hierarchical clustering of the treatments when sprouts were grown under a higher light intensity. Likewise, sprouts grown under the treatments at lower light intensity were clustered (Figure 5A). Additionally, the distribution of significant metabolite hits indicated that isoflavonoid biosynthesis was the most affected pathway (Figure 5B).

**Figure 6:**
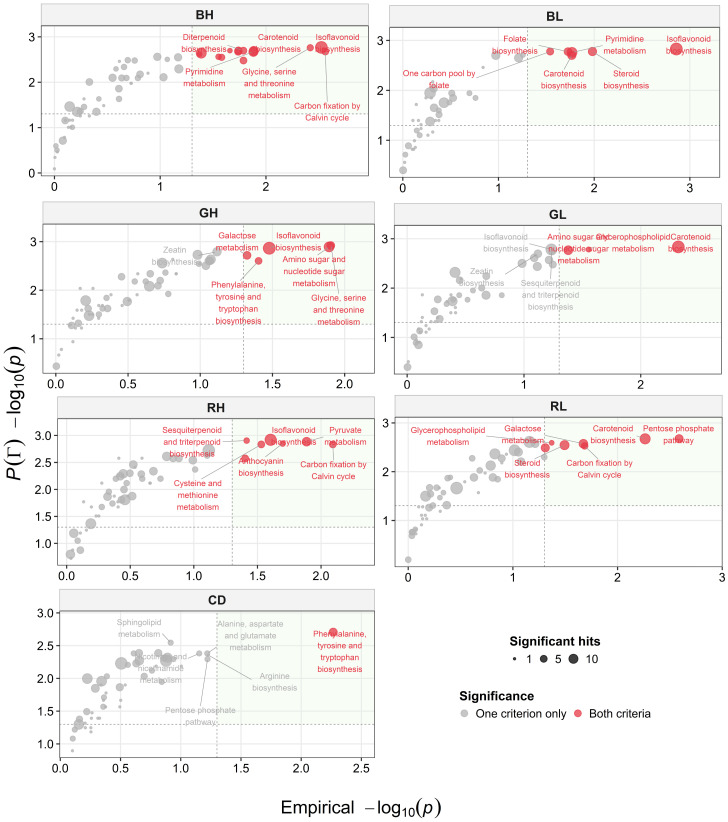
Scatter plots across all treatments compared to CS. The *X*-axis corresponds to −log_10_(p_empirical_) and the *Y*-axis to −log_10_(p_γ_). Each point represents a pathway; the size indicates the number of significant hits for that pathway, ranging from 1 to 10. The points that meet both criteria are in red inside the second quadrant, representing the more enriched pathways. Blue High (BH), Blue Low (BL), Control Dark (CD), Green High (GH), Green Low (GL), Red High (RH), and Red Low (RL) are the treatments corresponding to low (75 µmol·m^−2^·s^−1^) and high (275 µmol·m^−2^·s^−1^) light intensity.

When using the intersection of p-gamma and empirical p as a parameter, as plotted in log10 in Figure 6, anthocyanin biosynthesis was more significant under BH, RH, and CD. The highest number of significant metabolite hits was observed under the RH treatment. Isoflavonoid biosynthesis increased across all treatments, with 19 significant metabolite hits under BH.

**Figure 7:**
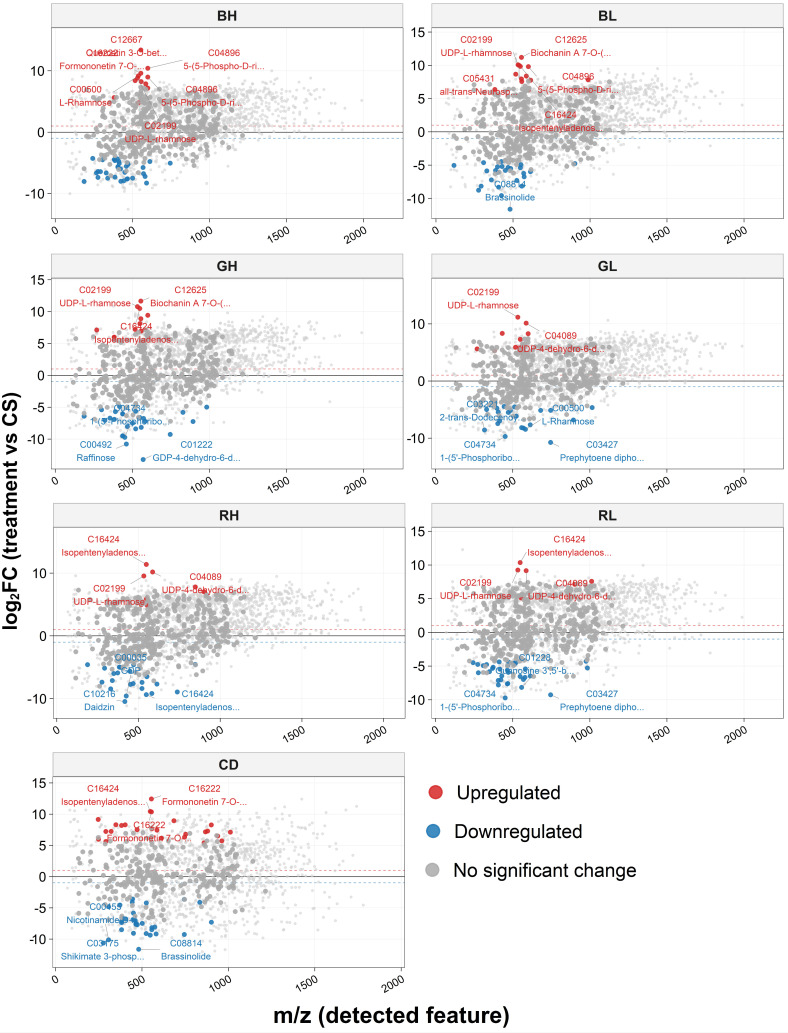
Global overview of all features. The *X*-axis uses log_2_FC, calculated as log_2_(PQN-normalized treatment) − log_2_(PQN-normalized CS). Horizontal dashed lines indicate the + and −1 log_2_FC threshold (2-fold change). Dots in red color (top 5 most enriched), blue (top 5 with lower enrichment ratios, most significantly, *p* < 0.05), and grey (the rest of the features) represent the metabolites found in the samples. Blue High (BH), Blue Low (BL), Control Dark (CD), Green High (GH), Green Low (GL), Red High (RH), and Red Low (RL) are the treatments corresponding to light treatments at low (75 µmol·m^−2^·s^−1^) and high (275 µmol·m^−2^·s^−1^) intensity.

The metabolomic response of chickpea sprouts under varying light conditions (wavelength and intensity) reveals significant insights into the plant’s adaptive mechanisms and metabolic shifts, causing upregulation and downregulation, as observed in Figure 7.

**Figure 8:**
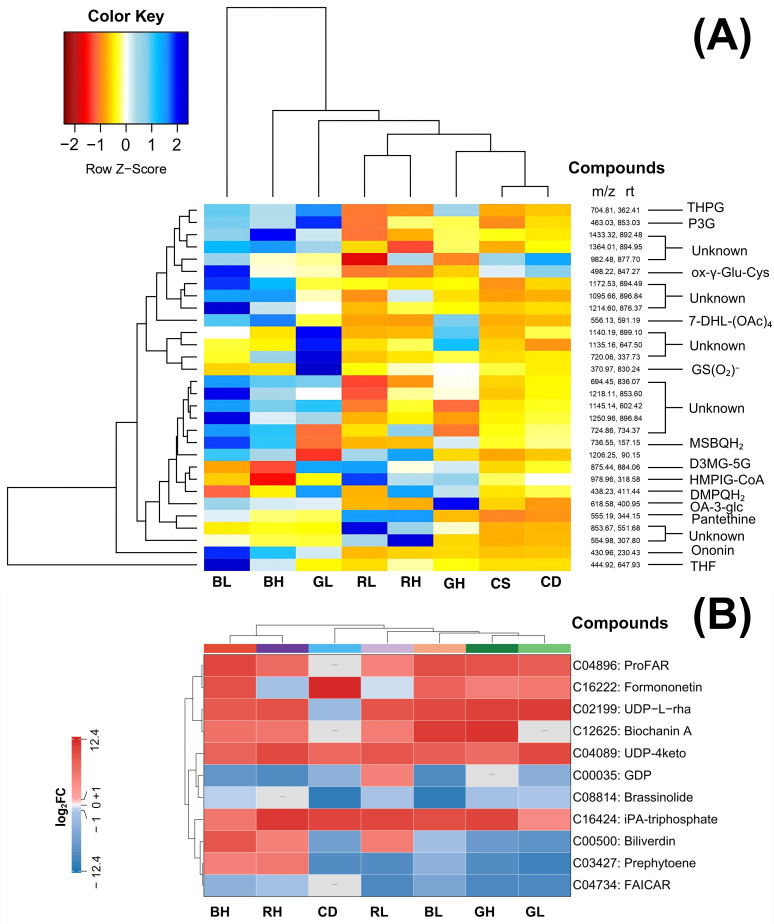
Metabolite concentration changes are induced by light in chickpea sprouts. In plot (**B**), Control Sun (CS) is not shown because all treatments were compared against it. (**A**) Metabolite profiles of chickpea sprouts exposed to the RGB light treatments at two intensities using HCA; all features were used, and only the top 30 were selected. Manual annotation was performed; it represents the heatmap of all features using the Z-score scale, while the second used the Mummichog algorithm. (**B**) Metabolite heatmap using Log_2_FC. The annotation was performed based on the peak list and the Mummichog algorithm. Blue High (BH), Blue Low (BL), Control Dark (CD), Green High (GH), Green Low (GL), Red High (RH), and Red Low (RL) are the treatments corresponding to light treatments at low (75 µmol·m^−2^·s^−1^) and high (275 µmol·m^−2^·s^−1^) intensity.

Heat maps showing metabolite concentration changes induced by light in chickpea sprouts are presented in Figure 8. Some metabolites with biological relevance putatively annotated were biochanin A 7-O-(6-O-malonyl-beta-D-glucoside) [M + Na]^+^, formononetin [M + K]^+^, proline at *m/z* 116 [M + H]^+^ (in all the treatments, significantly higher in CD and GH), and gamma-Aminobutyric Acid (GABA) at *m/z* 104 [M + H]^+^ (highest in CD). Furthermore, the following compounds were manually annotated based on the adduct and the *m/z* compared to the KEGG database: delphinidin 3-O-(6′′-O-malonyl)-β-D-glucoside-3′,5′-di-O-β-D-glucoside (D3MG-5G) [M + H]^+^, hydroxyMethylPent-3-IenylGlutaryl-CoA (HMPIG-CoA) [M + H]^+^, Ononin [M + H]^+^ Tetrahydropteroyltri-L-glutamate (THPG) [M + H]^+^, Peonidin 3-O-glucoside (P3G) [M + H]^+^, Oxidized gamma-glutamylcysteine (ox-γ-Glu-Cys) [M + H]^+^, S-Sulfinatoglutathione (GS(O_2_)^−^) [M + H]^+^, trans-Zeatin riboside triphosphate, Tetrahydropteroyltri-L-glutamate (THPG0) [M + H]^+^, 5-Methyltetrahydropteroyltri-L-glutamate (H3C-THPG) [M + H]^+^, Tetrahydrofolate (THF) [M + H]^+^, 7-Dehydrologanin tetraacetate (7-DHL-(OAc)_4_) [M + H]^+^, 2-Methyl-6-solanesylbenzene-1,4-diol (MSBQH_2_) [M + H]^+^, Demethylphylloquinol (DMPQH_2_) [M + H]^+^, Oleanolate 3-O-β-D-glucoside (OA-3-glc) [M + H]^+^, and Pantethine [M + H]^+^.

Chickpea sprouts grown under RGB light treatments exhibited higher metabolite concentrations and a shift in metabolic pathways compared with those grown under direct sunlight or in total darkness (Figure 8A). This was also previously described (Figure 5A,B). The blue light treatment yielded sprouts with the highest metabolite concentration globally, while CS showed the lowest. This suggests greater stress under blue light than in controls. Moreover, between controls, CD has a higher metabolite concentration than CS. On the other hand, when comparing the metabolite putative annotations against CS (positive control), after the Mummichog algorithm was applied, only metabolites from the legume source (*gmx_kegg*) were annotated, as observed in Figure 8B. Metabolite concentration across all treatments varied, with BH affecting metabolite concentration to a greater extent. A list of the annotated metabolites with *m/z* and RT is presented in Table S2.

## 4. Discussion

The observed changes in biomass may be correlated with the light wavelength and intensity. Biomass is reported to decrease with increasing light intensity [2]. Nevertheless, the decrease under BH and the increase in CS suggest that wavelength plays a fundamental role in biomass production, not only the intensity. The null changes in germination ratio suggest that chickpea seeds are light-neutral during sprouting; for instance, it has been reported that statistically significant differences occur only at the early stage (48 h) for this parameter [29].

The difference in stem length is due to etiolation in response to low light intensity; the rapid growth of the hypocotyl ensured that the apex reached the light before the seed ran out of nutrients [30], which explains why CD had the largest stem length. On the other hand, the difference observed under red light could be the result of hormonal interaction. A study reported a positive correlation between red light at 200 µmol·m^−2^·s^−1^ and hormones involved in stem elongation [31]. It was reported that red light at 100 erg/cm^2^/s can increase apical elongation in *Pisum sativum* L. through a hormonal effect (gibberellins), which may explain why higher red light levels produce larger stems (in contrast to the rest of the treatments) [32]. The information obtained from the morphological analysis suggests structural variation between the treatments. As discussed before, CD exhibits higher elongation, a thinner composition, and a reduced branching rate. These characteristics are an adaptive response to nutrient optimization, water acquisition, and light [33].

The previously described results correspond to the process in the chickpea sprouts and the plant carbon allocation. For instance, this process can be correlated with the specific photoreceptors of each wavelength. Blue light is known for maximizing the PSII quantum yield and carboxylation efficiency, while simultaneously activating the cryptochrome, and phototropin-mediated inhibition of cell expansion (therefore, also total dry biomass) can decline despite the high photosynthetic capacity [34]. This could explain why BH decreased and why CS was optimal. Moreover, in the CD phenotype, phyA reroutes seed-derived carbon through glycolysis and the TCA cycle to fuel axial elongation at the expense of radial thickening and branching [35]. Finally, the longer stems found under red light are consistent with phyB-mediated enhancement of gibberellin responsiveness rather than GA synthesis [36], which inevitably leads to outweighing the parallel wall-lignification response reported in legumes [37].

The increase in total phenolic composition is due to changes in physiological and metabolic processes. The metabolic route responsible for the formation of total phenolic compounds primarily involves the phenylpropanoid pathway and the flavonoid biosynthesis pathway [38,39]. Exposure to light variation is interpreted as an elicitation process that can alter the concentration and composition of total phenolic compounds. It is commonly reported that red and blue light enhance the accumulation of phenolic compounds by activating specific light receptors, such as phytochromes and cryptochromes, which regulate plant metabolism and growth; green light has not been studied that extensively [39]. Although blue and red light are the primary photoreceptors that can affect the photosynthetic system by stimulating the phenylpropanoid pathway, the results from this study suggest that low-intensity green light also promotes the production of phenolic compounds, indicating that photoreceptors alone may not be implicated. Nonetheless, the mechanism by which green light, specifically at 75 µmol·m^−2^·s^−1^, increases phenolic content remains unclear. Some studies suggest that deeper penetration of light improves photosynthetic efficiency, potentially leading to the overproduction of secondary metabolites [8]. In the same vein, it has been demonstrated that green light can deactivate photoreceptors typically activated by blue light, ultimately suppressing abscisic acid (ABA). In other words, exposure to low-intensity green light may trigger a photoprotective mechanism that leads to the overproduction of phenolic compounds. Ref. [40] found that LED light at 100 µmol·m^−2^·s^−1^ significantly affects genes in the phenylpropanoid pathway related to phenolic composition, which are highly expressed in the presence of red light, and are correlated with the increase in TPC as a response to abiotic stress [38,40]. In addition, ref. [8] demonstrated that seven-day germination of chickpea sprouts exposed to green and red light resulted in higher phenolic content, with green light increasing it by 30% compared to sunlight, and red light by 40%. The results obtained from this study confirm that the highest total phenolic content in chickpea sprouts is observed under red and green light treatments at high and low intensities, respectively, under the experimental conditions reported here.

Although BH was not the treatment with the highest phenolic content, the antioxidant activity results are considerably higher than those of the other treatments. This suggests that antioxidant activity depends not only on the total phenolic concentration but also on the specific metabolites present. Therefore, it is hypothesized that metabolites with high radical-scavenging capacity, including quercetin derivatives, contribute to this effect. Nevertheless, further experimentation is required as this study did not perform identification but rather putative annotation. Other authors found that LED blue light induced a significant increase in the antioxidant response [41]. In addition, the key role of blue light in kaempferol synthesis during legume germination has been identified [9]. The authors noted that blue light promotes ferulic acid accumulation, thereby elevating the antioxidant activity of legume sprouts. These results suggest that the increase in antioxidant activity under BH may correlate with the higher energy of blue light. Blue light has a shorter wavelength than the other treatments. In other words, higher energy enhances the photoprotection mechanism. High-intensity blue light increases reactive oxygen species (ROS) levels [42], thereby inducing a stress response that is more likely to be associated with production of secondary metabolites with high antioxidant activity. On the other hand, the difference in protein content between treatments suggests a positive response to light, consistent with findings in other research [43]. This suggests that green light can penetrate deeper into the sprout tissue, enhancing the embryonic photosynthesis. Also, another study examined the effects of green light on *Pisum sativum* L. and demonstrated that green light increased protein concentration along with red and blue light [4], making the results consistent across the literature and across the legume species. The mechanisms responsible for this have been identified as nitrate reduction and GS/GOGAT cycles when nitrogen is available, and channeling more carbon and nitrogen into amino acids and storage proteins via metabolic and signaling reprogramming.

The large difference between treatments at intensities of 75 μmol⋅m^−2^⋅s^−1^ and 275 μmol⋅m^−2^⋅s^−1^ suggests that metabolic reprogramming is not only due to wavelength variation but also to light intensity. The enrichment of glycine, serine, and threonine metabolism in all 275 μmol⋅m^−2^⋅s^−1^ groups (GH, BH, RH) is consistent with enhanced photorespiration activity since glycine and serine play a central role as photorespiratory intermediates; nevertheless, this may also reflect important changes in amino acid and one-carbon metabolism [44].

On the other hand, the increase in anthocyanin biosynthesis in RH and BH, but not in GH, may be correlated with the defense mechanism against light (which was not detected at low intensities). However, GH was ineffective in inducing anthocyanin biosynthesis. On the other hand, the data may suggest that the total absence of light (CD) acts as an elicitor rather than a stressor, modifying the amino acid biosynthetic pathway. Previous studies have reported a correlation between the anthocyanin biosynthesis pathway and blue and red pigments, acting as light defense (induced by CRY, PHY, UVR8, and HY5–MYB–bHLH–WD40 networks) [5]. This could explain why, under low intensities, the pathways are not perturbed.

Surprisingly, isoflavonoid biosynthesis was significantly affected in all conditions, with the highest enrichment under BH. Likewise, flavone and flavonol biosynthesis were significant across all treatments except under CD. This could be due to light signaling plus moderate photo-stress [8]. Also, carotenoid biosynthesis (CRY–HY5 and PSY/LCYB/CHYB induction) was greatly affected by the treatments, consistent with a previous report [45]. Carotenoids are highly light-responsive pigments involved in photoprotection. The data suggest that RGB light at two intensities modifies the pathways; additionally, the greater pathway perturbations in RH and BH may correlate with the previously identified higher TPC and antioxidant activity.

The metabolite profile with higher compound concentrations was tentatively assigned to the blue treatments, suggesting greater abiotic stress due to blue light exposure. It has been reported that blue light activates specific metabolic pathways, including those responsible for flavonoid and anthocyanin synthesis [46]. The results reveal a key change in metabolism, driven primarily by light quality and, secondarily, by intensity. Blue light induces a comprehensive reprogramming of metabolomic pathways in chickpea sprouts. That blue light can change up to 25% of the gene expression, with most of these changes mediated by CRY1 and CRY2, has been previously reported [47]. It has been reported that blue light activates cryptochromes, phototropins, and the ZTL/FKF1/LKP2 family signaling pathways and can induce metabolic stress, which is directly correlated with the synthesis of multiple metabolites [46,47,48]. Additionally, red light treatments increase metabolites associated with the phenylpropanoid pathway (Peonidin 3-O-glucoside, D3MG-5G), suggesting that light conditions are perceived as mild elicitors rather than stressors [38]. Anthocyanins such as D3MG-5G and Peonidin 3-O-glucoside were putatively annotated as responding to red, green, and blue light, suggesting a change in metabolic pathways. Nevertheless, anthocyanins can be produced under sunlight and even under total darkness, although the anthocyanin concentration is significantly lower than that of sprouts treated with RGB. It has been reported that in many plant species (with limited studies in legumes), light quality and quantity can affect anthocyanin accumulation [49]. Their role consists of defending against viral, bacterial, and fungal activity, absorbing excess visible and UV irradiance, and scavenging ROS under abiotic stress [49]. The highest D3MG-5G (delphinidin 3-O-(6′′-O-malonyl)-β-D-glucoside-3′,5′-di-O-β-D-glucoside) concentration was obtained under red light at 75 µmol·m^−2^·s^−1^, followed by green light at the same intensity. In comparison, the highest concentration of Peonidin 3-O-glucoside was recorded under green light at 75 µmol·m^−2^·s^−1^, followed by blue light at the same intensity. Ref. [50] determined that blue and red light affect anthocyanin accumulation, increasing it via CRY2/COP1 and HY5, or via ABA signaling. Additionally, in a study with three varieties of mung bean sprouts, it was found that red light significantly increased delphinidin concentration compared with white and blue light (which had the lowest concentration) [51]. On the other hand, even though not many studies have been conducted on the effect of green light on anthocyanin accumulation, it has been determined that green light can act as a promoter for photosynthesis due to the increase in the development of plastids and the metabolism inside them, which leads to anthocyanin accumulation [52].

Several putative metabolites showed increased treatment-associated changes relative to Control Sun. The following results are expressed as log_2_FC values, and treatments are indicated: biochanin (4.82, RH); cyanidin 3-O-glucoside (5.83, GL). These metabolites are associated with total phenolic compounds across all treatments. For antioxidant activity, the compounds related are quercetin 3-O-beta-D-glucosyl-(1->2)-beta-D-glucoside (13.38, BH); for protein, molybdopterin (1.64, GH); furthermore, red chlorophyll catabolite (8.94, CD), and trans-Zeatin riboside triphosphate (6.43, CD) were also found (Supplementary Table S3). These associations are presented as hypotheses rather than confirmations, due to the putative (MSI level 3) condition of the annotations. Considering that caveat, the data are consistent with red light at 275 μmol⋅m^−2^⋅s^−1^ (and low-light-intensity green light) favoring the accumulation of phenolic compounds. At the same time, the significant difference in antioxidant activity of BH may be partially explained by quercetin 3-O-beta-D-glucosyl-(1->2)-beta-D-glucoside accumulation. On the other hand, molybdopterin was detected under GH. As the molybdenum-containing cofactor of nitrate reductase and other molybdoenzymes required for nitrate assimilation, its accumulation may be compatible with a higher protein content [53]. The presence of a red chlorophyll catabolite is consistent with the absence of pigmentation in CD [54]. Furthermore, the data showed that the cytokinin trans-Zeatin riboside triphosphate was at a high concentration under CD, consistent with greater stem elongation. However, more studies are required, as specific proteins are also involved in stem elongation, including A0A1S2XHQ9 (Elongation factor 1-alpha) [55]. Confirming any of these links would require targeted techniques, standards-based quantification, and correlation with the corresponding phenotype.

Final seed quality is sensitive to factors such as heat, chilling, and water deficit. To our knowledge, no study has examined seed filling in chickpea or other legumes under RGB LED light. Nonetheless, these results (7-day sprouts), as a first approach, help to understand metabolite changes; however, more studies should be conducted to define seed quality in the final growth stage; in other words, more insight is needed to establish whether the metabolic and protein changes observed in this early stage, as conducted in this study, will persist into seed filling or affect final seed composition and quality. This remains an open question and is proposed as a direction for future research. Furthermore, the study was performed using a 3D ion-trap mass spectrometer, which provides nominal rather than accurate mass, making compound assignments putative (MS1 level 3). In this scenario, high-resolution MS/MS fragmentation and quantification with metabolite-specific standards would confirm the candidate metabolites and pathways highlighted here.

## 5. Conclusions

This study demonstrated that light intensity and wavelength significantly influenced the protein content and secondary metabolite composition of the chickpea sprouts’ embryonic axis and modified metabolic pathways, with greater effects under blue light. High-intensity red light significantly increased total phenolic content, indicating robust activation of the phenylpropanoid pathway. Green light at high intensity doubled the protein content, suggesting enhanced nitrogen assimilation and protein synthesis. High-intensity blue light significantly elevated antioxidant activity, coinciding with a higher abundance of antioxidant metabolites in the embryonic axis. This research demonstrated that metabolic pathway perturbation is light-wavelength and intensity dependent, with higher modification in isoflavonoid biosynthesis under blue and red light and high intensity. These findings underscore the potential of light manipulation to enhance the nutritional and functional qualities of chickpea sprouts, contributing to food security and human health. Further research is needed to elucidate the molecular and genetic mechanisms underlying these metabolic changes and to explore the bioavailability and health impacts of the induced compounds. Additionally, it is suggested that light effects be explored across different chickpea growth stages up to the final seed, which will be fundamental to producing food with higher levels of beneficial compounds for human health. Finally, future work must analyze the differences between the embryonic axis and the cotyledons, as separate tissues and as a group.

## Figures and Tables

**Table 1:** Biochemical profile of chickpea sprouts embryonic axis.

Treatment	TPC (mg GAE/g DW)	Antioxidant Activity (µmol TE/g DW)	Protein (mg BSA-eq/g DW)
RH	20.4 ± 1.2 ^a^	44.8 ± 3.2 ^b^	14.0 ± 0.9 ^e^
RL	17.4 ± 1.0 ^b^	28.4 ± 0.8 ^c^	20.0 ± 1.3 ^cd^
GH	13.3 ± 0.5 ^c^	31.1 ± 1.7 ^c^	26.4 ± 0.7 ^a^
GL	20.5 ± 0.2 ^a^	21.2 ± 0.2 ^d^	24.5 ± 1.6 ^b^
BH	18.1 ± 0.2 ^b^	88.9 ± 2.6 ^a^	21.3 ± 0.8 ^c^
BL	15.6 ± 1.4 ^bc^	29.1 ± 2.9 ^c^	25.5 ± 1.0 ^ab^
CD	6.3 ± 0.7 ^d^	25.9 ± 1.3 ^c^	12.7 ± 0.3 ^f^
CS	6.7 ± 0.5 ^d^	50.5 ± 1.0 ^b^	15.3 ± 0.4 ^e^

Total phenolic content (TPC) of chickpea sprouts; GAE refers to gallic acid equivalents. Antioxidant activity expressed as Trolox equivalents (TE) per gram of dry weight (DW). Soluble protein content was expressed as BSA (Bovine Serum Albumin) equivalents per gram of dry weight. Treatments correspond to red (R), green (G), and blue (B) light at two intensities, 75 (low, L) and 275 (high, H) PPFD (µmol·m^−2^·s^−1^), yielding six treatments: RL, RH, GL, GH, BL, and BH. CS and CD were the controls, positive and negative, respectively. Values were expressed as mean ± standard error of the mean (SEM; *n* = 3 biological replicates). Values were normalized per gram of dry weight. The superscript letters indicate statistically significant differences among the treatments, obtained using Tukey’s Honestly Significant Difference (HSD) test at 95% confidence (*p* < 0.05).

## Data Availability

The data presented in this study are available on request from the corresponding author. The data presented in this study are available at Zenodo: https://doi.org/10.5281/zenodo.21043444. The .mzML files and other important files are contained in MassIVE with the accession number MSV000102307, https://doi.org/doi:10.25345/C52J68J88.

## Supplementary Materials

The following supporting information can be downloaded at: https://www.mdpi.com/article/10.3390/foods15142578/s1, Supplementary Materials S1. It contains Figure S1. Environmental conditions during the experiment, Figure S2. Daily light integral (DLI) from the LED light source and sunlight, Figure S3. Greenhouse details, Code S1. Code used for monitoring the greenhouse conditions across the experiment period, Figure S4. Greenhouse configuration, Table S1. Effects of Red-Green-Blue (RGB) light at two intensity levels on the morphological characteristics of seven-day-old, germinated chickpea (*Cicer arietinum* L.) sprouts, and Table S2. Putative compounds. Furthermore, Supplementary Materials S2. Table S3 contains the list of features detected across all treatments. Scripts and peak lists can be downloaded at Zenodo: https://doi.org/10.5281/zenodo.21043444. The .mzML files and other important files are contained in MassIVE with the accession number MSV000102307, https://doi.org/doi:10.25345/C52J68J88.
