# Supplementary material for: Light Intensity and Wavelength Modulate Antioxidant Secondary Metabolites in Embryonic Axis of Chickpea Sprouts
Source: Foods. 2026 Jul 22;15(14):2578. doi: 10.3390/foods15142578 (PMC13409064; doi:10.3390/foods15142578)
Supplement: Supplementary file 1 [file foods-15-02578-s001.zip › foods-4377374-supplementary/foods-4377374-supplementary.pdf]

Light Intensity and Wavelength Modulate Antioxidant Secondary Metabolites in Embryonic Axis of Chickpea Sprouts

Luis F. Pérez-Hernández [1], Robert Winkler [2] and Marco A. Mata-Gómez [1,*]

Supplementary Materials S1

This supplementary material was built to provide information about the greenhouse construction and operation. In addition, this Supplementary Material also contains results from the morphological analysis of the sprouts.

Experiment location

The experiment for biomass production of the chickpea sprouts was done in Puebla, Mexico (19°01'05.0"N 98°14'32.4"W), elevation 2, 135 m above sea level. It was conducted between 2024 and 2025, from March to May (in both cases). The weather conditions from Puebla city (Figure S1 and S2) were contrasted with those reported by the government. The analysis showed high consistency across the conditions.

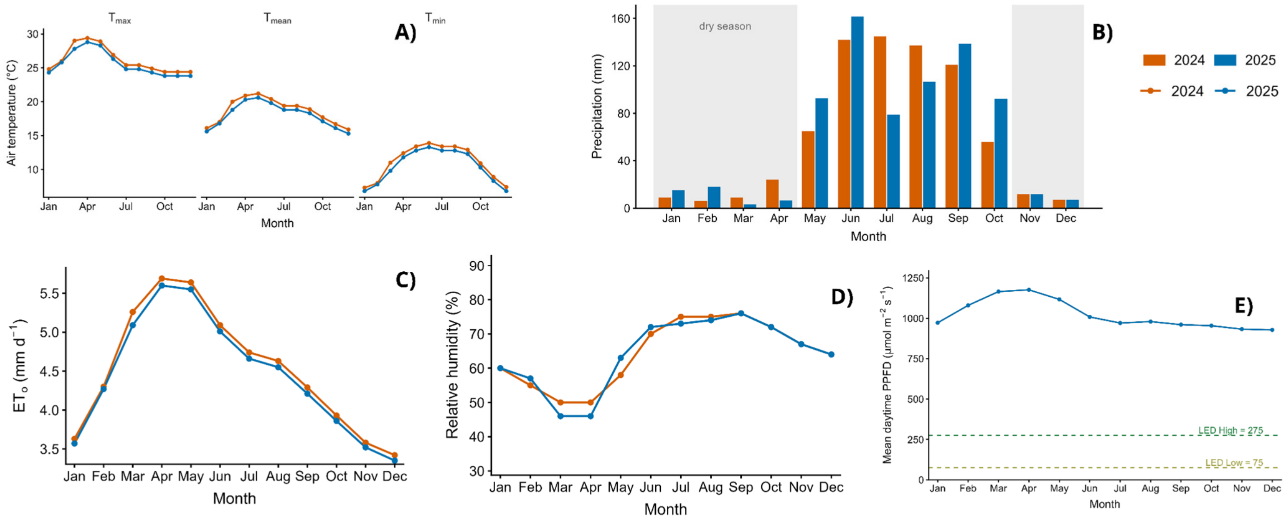

Figure S1. Environmental conditions during the experiment. **A)** Temperature variation during 2024 and 2025. **B)** Precipitations in Puebla city, the grey shading represents the dry season. **C)** Evapotranspiration during the experimental years. **D)** Relative humidity of the environment. **E)** Light intensity comparison from LED treatments and the control sun.

Daily light integral (DLI) was calculated using the following equation: $DLI = PPFD \cdot Photoperiod \cdot 3600/1000{,}000$.

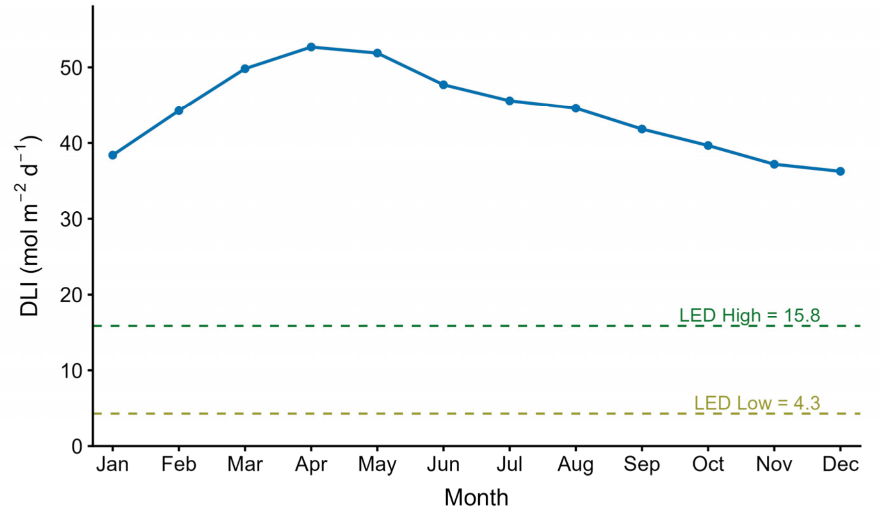

Figure S2. Daily light integral (DLI) from the LED light source and sunlight (average across the two years), the blue light corresponds to the data of the sunlight.

Light system implementation

Light was provided by an RGB light system (Tecnoled, model ML-REF-RGB). The photoperiod was 16 hours of light and 8 hours of total darkness. The light regulation was automated using a timer (TEMP-08E, Steren, Mexico City, Mexico) and was constantly monitored to ensure the photoperiod was accurate.

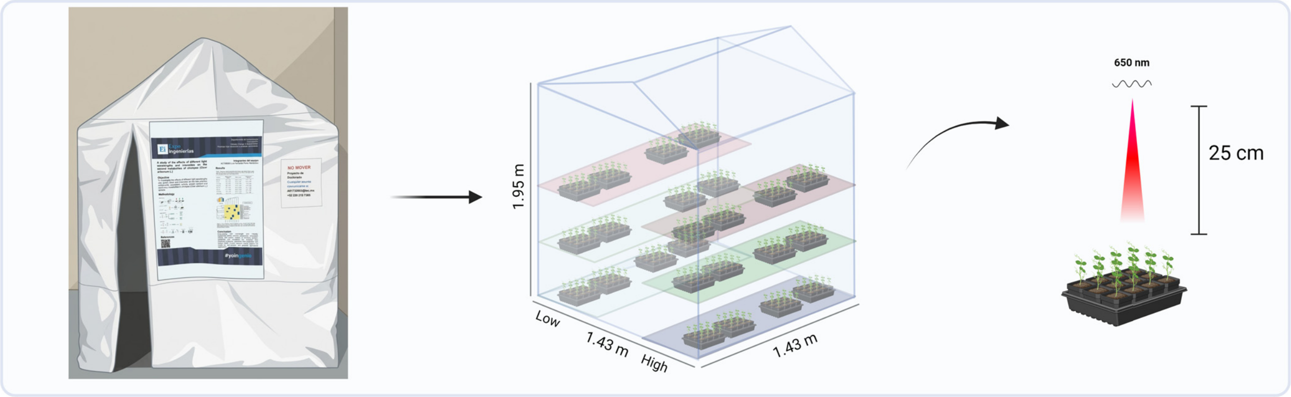

Figure S3. Greenhouse details. The first part corresponds to the real structure of the greenhouse. The second part corresponds to the internal structure, and the third to a zoom of how the light was administered to the sprouts.

The following sensors were used for monitoring the greenhouse: temperature and humidity (DHT11), Capacitive Anticorrosive Soil Moisture (AR1188), and light (TSL2561), and the code was programmed in Arduino.

```
#include <Wire.h>
#include <SPI.h>
#include <SD.h>
#include <DHT.h>
#include <RTClib.h>
#include <Adafruit_Sensor.h>
#include <Adafruit_TSL2561_U.h>


#define DHTPIN 2
#define DHTTYPE DHT11
```

```
#define SOIL_PIN A0
#define SD_CS 10

DHT dht(DHTPIN, DHTTYPE);
RTC_DS3231 rtc;
Adafruit_TSL2561_Unified tsl = Adafruit_TSL2561_Unified(TSL2561_ADDR_FLOAT, 12345);

int valorSeco = 800;
int valorMojado = 350;

void setup() {
  Serial.begin(9600);
  dht.begin();

  if (!rtc.begin()) {
    Serial.println("No se detecto RTC");
    while (1);
  }

  if (!SD.begin(SD_CS)) {
    Serial.println("No se detecto microSD");
    while (1);
  }

  if (!tsl.begin()) {
    Serial.println("No se detecto TSL2561");
    while (1);
  }
```

```
  tsl.enableAutoRange(true);
  tsl.setIntegrationTime(TSL2561_INTEGRATIONTIME_402MS);

  Serial.println("Sistema iniciado");
}

void loop() {
  DateTime now = rtc.now();

  char filename[20];
    sprintf(filename,   "%04d-%02d-%02d.csv",   now.year(),   now.month(),
now.day());

  bool nuevoArchivo = !SD.exists(filename);

  File archivo = SD.open(filename, FILE_WRITE);

  if (archivo) {
    if (nuevoArchivo) {
      archivo.println("Fecha,Hora,Temperatura_C,Humedad_Ambiental_%,Humedad_
Suelo_%,Luz_lux");
    }

    float temperatura = dht.readTemperature();
    float humedadAmbiental = dht.readHumidity();

    int lecturaSuelo = analogRead(SOIL_PIN);
    int humedadSuelo = map(lecturaSuelo, valorSeco, valorMojado, 0, 100);
    humedadSuelo = constrain(humedadSuelo, 0, 100);
```

```
sensors_event_t event;
tsl.getEvent(&event);

float lux = event.light ? event.light : 0;

archivo.print(now.year());
archivo.print("-");
archivo.print(now.month());
archivo.print("-");
archivo.print(now.day());
archivo.print(",");

archivo.print(now.hour());
archivo.print(":");
archivo.print(now.minute());
archivo.print(":");
archivo.print(now.second());
archivo.print(",");

archivo.print(temperatura);
archivo.print(",");
archivo.print(humedadAmbiental);
archivo.print(",");
archivo.print(humedadSuelo);
archivo.print(",");
archivo.println(lux);

archivo.close();
```

```
    Serial.println("Datos guardados en SD");
  } else {
    Serial.println("Error al abrir archivo");
  }

  delay(17280000);
}
```

Code S1. Code used for monitoring the greenhouse conditions across the experiment period.

The greenhouse was installed under a wall-less polyethylene canopy (Annex 1). To ensure total darkness during the day, a dual-layer film (70% opacity) was implemented in the greenhouse (one on the external surface and one on the internal). Additionally, the interior surfaces were coated with a black matte finish and reinforced with black polyethylene to prevent environmental light contamination (PPFD was under the limit of detection). The LED light treatment was set to start only during the late afternoons and nights to reduce heat accumulation. Each treatment was isolated from the others using a light-blocking material, preventing any light mixing that could disturb the treatments (Figure S4).

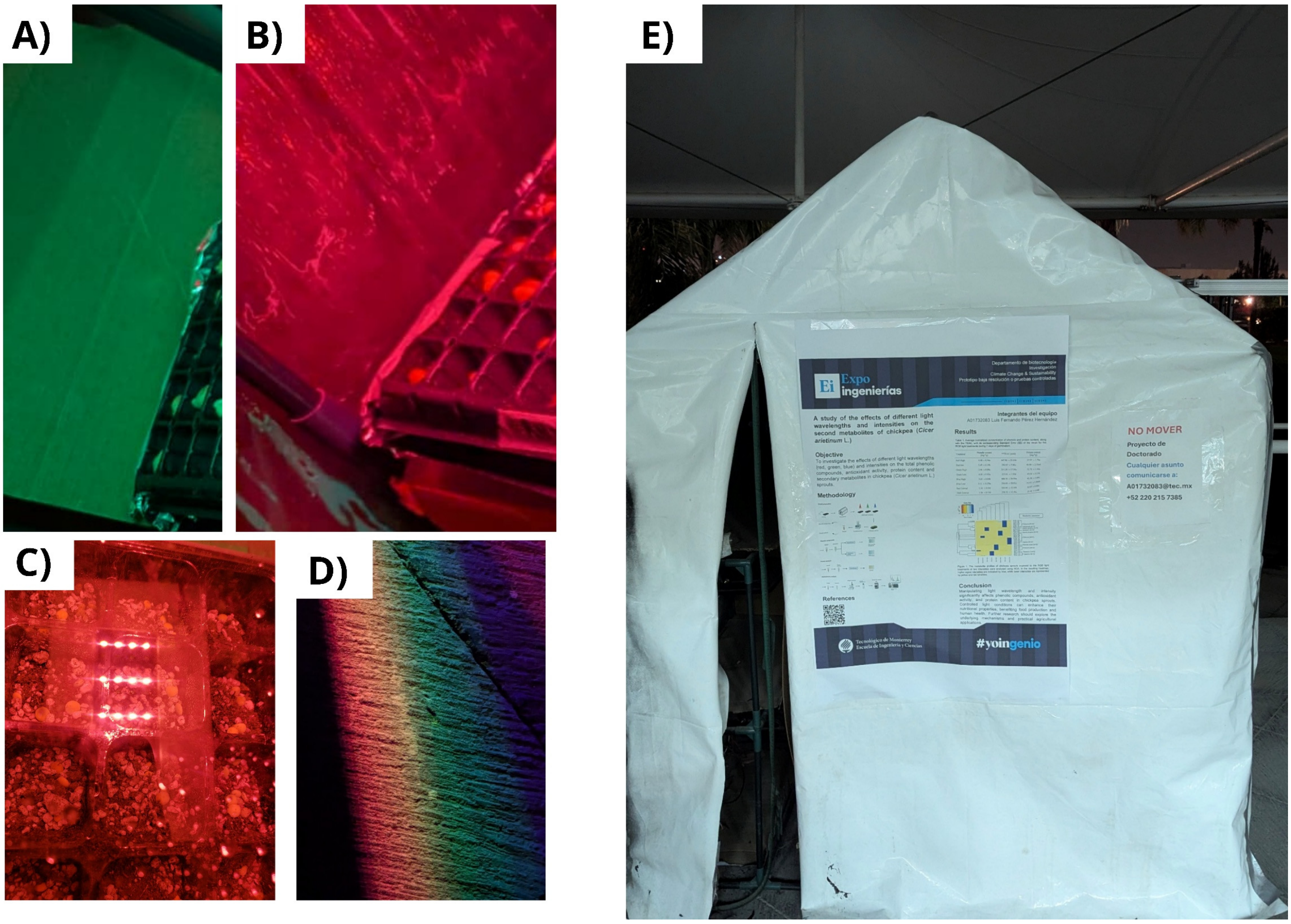

Figure S4. **A)** Green light showing how it was managed to reduce light contamination. **B)** Red light, the pots were considered first as open; however, **C)** it was decided to use the cover (light measurement was done inside) to keep the relative humidity. **D)** light combination when not handled correctly. **E)** Greenhouse photography. The aperture was handled by a zip.

Table S1. Effects of Red-Green-Blue (RGB) light at two intensity levels on the morphological characteristics of seven-day-old, germinated chickpea (*Cicer arietinum* L.) sprouts.

Treatment	Fresh weight (g)	Germination ratio (%)	Average length of sprout (cm)	LRBD (tips·cm^{-1})	Depth (cm)	Width to Depth Ratio	Average Diameter (cm)	Volume (cm^3)	Steep Angle Frequency
RH†	0.90 ± 0.40 ab*	58 ± 24 a	2.48 ± 0.12 bc	6.46 ± 0.68 c	1.74 ± 0.14 b	0.32 ± 0.02 c	0.057 ± 0.004 b	0.011 ± 0.0020 c	0.58 ± 0.04 b
RL‡	0.91 ± 0.08 ab	63 ± 3 a	2.10 ± 0.06 bc	3.81 ± 0.38 d	1.84 ± 0.14 b	0.19 ± 0.01 d	0.090 ± 0.01 a	0.023 ± 0.0041 b	0.82 ± 0.05 a
GH	0.73 ± 0.38 ab	55 ± 16 a	0.97 ± 0.01 d	7.22 ± 0.77 bc	0.40 ± 0.03 c	0.44 ± 0.02 b	0.058 ± 0.004 b	0.002 ± 0.0004 e	0.48 ± 0.02c
GL	1.18 ± 0.59 ab	75 ± 7 a	1.93 ± 0.94 cd	14.01 ± 3.92 a	0.64 ± 0.15 c	0.23 ± 0.05 d	0.025 ± 0.001 d	0.001 ± 0.0002 e	0.51± 0.04 bc
BH	0.53 ± 0.43 a	53 ± 21 a	1.18 ± 0.43 d	7.62 ± 1.27 bc	0.39 ± 0.06 c	0.83 ± 0.15 a	0.048 ± 0.003 c	0.002 ± 0.0003 e	0.31 ± 0.02 d
BL	1.14 ± 0.51 ab	67 ± 22 a	3.34 ± 0.37 b	8.37 ± 1.31 b	0.99 ± 0.10 c	0.23 ± 0.02 d	0.026 ± 0.002 d	0.002 ± 0.0003 e	0.57 ± 0.05 b
CS	1.81 ± 0.08 b	86 ± 7 a	2.50 ± 0.28 bc	10.82 ± 1.58 b	1.09 ± 0.11 c	0.18 ± 0.02 d	0.056 ± 0.005 b	0.022 ± 0.0046 b	0.59 ± 0.05 b
CD	1.39 ± 0.33 ab	70 ± 10 a	5.72 ± 0.37 a	2.80 ± 0.32 e	3.97 ± 0.35 a	0.18 ± 0.01 d	0.060 ± 0.005 b	0.008 ± 0.0015 c	0.79 ± 0.05 a

† = High intensity (275 μmol·m^{-2}·s^{-1}), ‡ = Low intensity (75 μmol·m^{-2}·s^{-1}), every type of LED light has the same intensities, which are H and L. *abcd = letter variation indicates significant differences between treatments ($p < 0.05$).

RH = Red High, RL = Red Low, GH = Green High, GL = Green Low, BH = Blue High, BL = Blue Low, CS = Control Sun, and CD = Control Dark. The fresh weight, germination ratio, and average length were measured because they are indicators of sprout vigor. These parameters, along with others, help assess edible yield and early root system architecture, which are relevant growth indicators.

Table S2. Putative compounds.

KEGG ID	Compound	Adduct	*m/z*	RT (s)
C04896	ProFAR	$[M+Na]^{+}$	600.00	657.9
C16222	Formononetin	$[M + K]^{+}$	555.08	810.5
C02199	UDP−L−rha	$[M−H2O+H]^{+}$	532.98	680.4
C12625	Biochanin A	$[M+Na]^{+}$	555.14	810.7
C04089	UDP-4keto	$[M+K]^{+}$	586.85	760.3
C00035	GDP	$[M−H2O+H]^{+}$	425.59	753.5
C08814	Brassinolide	$[M+H]^{+}$	481.07	836.3
C16424	iPA-triphosphate	$[M−CO+H]^{+}$	548.16	771.6
C00500	Biliverdin	$[M−CO2+H]^{+}$	539.08	154.8
C03427	Prephytoene	$[M+Na]^{+}$	745.42	842.2
C04734	FAICAR	$[M+HCOOK]^{+}$	450.82	553.2
